# Esophageal cancer in Mozambique: should mycotoxins be a concern?

**DOI:** 10.11604/pamj.2019.33.187.18295

**Published:** 2019-07-11

**Authors:** Jotamo Come, Edgar Cambaza, Rita Ferreira, José Manuel Correia da Costa, Carla Carrilho, Lúcio Lara Santos

**Affiliations:** 1 Department of Surgery, Maputo Central Hospital, Maputo, Mozambique; 2 Department of Biological Sciences, Faculty of Sciences, Eduardo Mondlane University, Maputo, Mozambique; 3 QOPNA-Química Orgânica, Produtos Naturais e Agroalimentares, Departamento de Química, Aveiro University, Aveiro, Portugal; 4 Center for the Study of Animal Science, ICETA, University of Porto and INSA-National Health Institute Dr. Ricardo Jorge, Porto, Portugal; 5 Department of Pathology, Faculty of Medicine, Eduardo Mondlane University, Maputo Central Hospital, Maputo, Mozambique; 6 Experimental Pathology and Therapeutics Research Group, Surgical Oncology Department, Portuguese Oncology Institute, Porto, Portugal; 7 ONCOCIR, Education and Care in Oncology, Lusophone Africa

**Keywords:** Mycotoxyns, esophageal cancer, Mozambique

## Abstract

Fumonisin B1 (FB1) is a mycotoxin frequently found in agricultural commodities. The toxin poses a considerable risk for human and animal health. FB1 is among several mycotoxins produced by Fusarium spp. contaminating virtually any cereal and other Poaceae. Their intracellular action includes the promotion of oxidative stress through the generation of reactive oxygen species (ROS) that damage biomolecules such as DNA. These toxic effects were observed in vivo and in vitro. However, the association between esophageal lesions and oxidative stress induced by FB1. Studies in China, Iran and South Africa showed higher exposure to fumonisins in areas with higher risk of esophageal cancer (EC). Exposure to mycotoxins may be inevitable in Mozambique. How mycotoxins, particularly fumonisins from the contaminated food, can be associated with the emergence of EC in Mozambique? Herein, we revise the literature and present some pieces of evidence in order to highlight the burden of mycotoxins and to provide evidence-based considerations for the stakeholders involved in the management of the EC agenda in Mozambique. The information presented herein supports the need to implement novel and/or to revisit the existent detoxification methods to reduce the global burden of mycotoxins and its outcomes in health management.

## Essay

Cancer of esophagus is a serious health problem in sub-Saharan Africa and it is associated with high lethality [1]. Carcinogenesis of esophageal cancer (EC) is still largely unknown in sub-Saharan Africa. Several individual factors have been considered but the main cause is most likely multifactorial [2]. EC, mainly squamous cell carcinoma, is highly prevalent in Western Kenya, especially among members of the Kalenjin community, who reside in the northern and southern areas of the rift valley [3]. According to Kigen *et al*. (2017), the most plausible causes of the high incidence of EC among the Kalenjin community are mycotoxins, particularly fumonisins from the food chain resulting from poor handling of cereals [4]. Aflatoxins and fumonisins are mycotoxins contaminating a large fraction of the world's food, including maize, cereals, groundnuts and tree nuts [5]. Contamination is due to high-level chronic exposure [6]. This is particularly true in subsistence farming communities where regulations to control exposure are either non-existent or practically unenforceable [7].

### Esophageal cancer in Mozambique

Mozambique has a high rate of EC. According to previous studies, EC is the 4^th^ most incident malignant tumor in Maputo in both genders [8]. A study of 522 consecutive cases of EC diagnosed and treated at the Maputo Central Hospital revealed that most patients were female (n=291, 55.7%), and born in the southern region of the country (n=418, 80.1%) where the consumption of maize in food and in fermented beverages is high [9]. The prognosis is highly undesirable as the median survival time was 3.5 months for all patients [9]. Therefore, it is necessary to implement a nationwide esophageal cancer program in Mozambique encompassing the detection of risk factors and the implementation of early diagnosis programs. Can mycotoxins play a role in the malignant transformation process of the esophagus in Mozambique? Kigen *et al*. (2017) suggest that mycotoxins, particularly fumonisins, combined with traditional alcohol, dietary deficiencies and viral infections acting synergistically are risk factors for EC in the Western Kenya [4]. So, one can suspect of a similar effect in Mozambique once food habits are similar.

### Fusarium verticillioides and carcinogenesis

**Figure 1:**
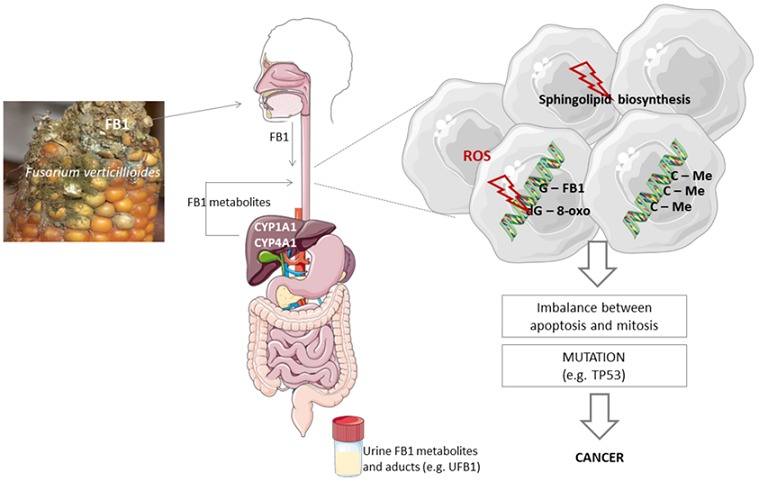
Overview of the putative molecular pathways involved in EC induced by FB1. (C-Me - DNA hypermethylation; CYP - cytochrome P450; dG-8-oxo - 8-Oxo-2’-deoxyguanosine; FB1- fumonisin B1; UFB1- urinary fumonisin B1)

Fusarium verticillioides, a mold that grows mostly on maize, has the ability to produce Fumonisin B1 (FB1). FB1 is a toxic secondary metabolite linked to EC and neural tube defects in humans and lung edema in swine and leukoencephalomalacia in equines [10]. Their intracellular action, favouring oxidative stress and the generation of reactive oxygen species (ROS), sustain their toxic effects observed in vivo [11,12] and in vitro [13,14]. Mycotoxins have a strong tendency and ability to penetrate the human and animal cells and reach the cellular genome where it causes a major mutagenic change in the nucleotide sequence, which leads to strong and permanent defects in the genome (adduct formation targeting guanine bases, which induces G→T transversions at codon 249 in TP53) [15,16]. FB1 might also disrupt sphingolipid metabolism therefore impairing the balance between apoptosis and mitosis [17]. These defects will eventually be transcribed, translated and lead to the development of cancer (Figure 1). FB1 is a known animal carcinogen and has been shown to cause tumors of the liver and kidney in mice and rats [18]. Likewise, chronic dietary exposure to FB1 (≥50 ppm) is carcinogenic to rodents: hepatocarcinogenic in male BD IX rats and female B6C3F1 mice and nephrocarcinogenic in male F344 rats [19,20]. The weight of evidence indicates that the mechanism of carcinogenesis is epigenetic and related to compensatory cell proliferation accompanying apoptosis [21]. Fumonisin was categorized as a Group 2B carcinogen by the International Agency for Research on Cancer (CIRC) [22].

### Fumonisins and esophageal cancer

So far, evidence for human carcinogenicity of fumonisins is circumstantial and limited. Yet, studies in China, Iran, and South Africa showed higher exposure to fumonisins in areas with higher risk of EC [23]. Consumption of contaminated maize has been associated with an elevated risk of EC in the Transkei region in South Africa and China [24,25]. Maize consumption by different age groups in these communities was measured in Mbizana (formerly known as Bizana) and Centane magisterial areas of the former Transkei region of the eastern cape province of South Africa, an area of high EC incidence [25]. Mean fumonisin exposures in all age groups were above the provisional maximum tolerable daily intake according FAO/WHO Expert Committee on Food Additives. Mwalwayo and Thole (2016) observed that populations in the rural areas of Malawi, where the incidence of EC is also high, may be at a high risk of exposure to unacceptably high levels of aflatoxins and fumonisins, according to the *Codex Alimentarius*. This seems more preoccupant in the Chikhwawa and Machinga districts from the southern part of the country where relatively high levels of both aflatoxins and fumonisins were observed [26]. This region is bordered by Mozambique. However, the only case-control study on fumonisin exposure in relation to EC risk was conducted in Linxian, China, and no association between exposure and risk was found [27]. Urinary fumonisin B1 (UFB1) was the exposure biomarker assayed once it offers an integrated estimation of exposure from all sources for either aflatoxin or fumonisin [5]; however, the results were inconclusive. UFB1 has been measured in human samples in regions with known high exposure to dietary fumonisins [28]. In general, statistically significant relationships between UFB1 and either estimated or measured FB1 intakes were reported; however, the data indicate that urinary measure was only moderately reflective of the intake level [29]. According to Liu *et al*. (2016), mutation analysis revealed common signatures across esophageal cancer samples from Malawi patients associated with aging, cytidine deaminase activity, and a third signature of unknown origin. Signatures of mycotoxins were notably absent [30]. Therefore, epidemiologic studies are needed to establish or refute any association between fumonisins and EC.

### Mycotoxin in Mozambique

Aflatoxins B1 (AFB1) and G1 (AFG1) have been found in Mozambican commodities, especially groundnuts and maize [31]. Casadei found aflatoxins in food samples from different areas of Mozambique, though it was almost 40 years ago [32]. Yet, more recent evidences reported by Sineque *et al*. (2017) and Zuza *et al*. (2018) demonstrate that aflatoxin exposure is still a major issue in Mozambique [33,34]. According to Cambaza *et al*. (2018a,2018b), the highest prevalence of aflatoxins contamination was found in Nacala, followed by Maputo city. Inhambane and Nampula also had high aflatoxin levels in their foods [31,32]. Warth *et al*. (2012) studied mycotoxins in food and feed from Burkina Faso and Mozambique and observed that FB1 concentration in maize were higher in Mozambique (92% incidence, median = 869 µg/kg) than in Burkina Faso (81% incidence, median = 269 µg/kg). Their samples were purchased in markets from Nampula city [35]. New strategies for fighting food contamination by mycotoxins are urgently needed [36].

The working group from IARC regarding food contamination with fumonisin B1 performed the present evaluation: there is inadequate evidences in humans for the carcinogenicity of fumonisins. However, there is sufficient evidence from experimental animals for the carcinogenicity of FB1. Thus, FB1 is possibly carcinogenic to humans (Group 2B). These subjects are usually exposed to other risk factors among which are indoor air pollution caused by cooking with charcoal. So, the combination of these risk factors may be at the genesis of the high esophageal cancer rates in Mozambique. We believe that it is necessary to study these issues, to educate the population, to move beyond ecological evidence and to promote food security with cost-effective measures.

## Competing interests

The authors declare no competing interests.
